# Application of Motion Sensors for Beam-Tracking of Mobile Stations in mmWave Communication Systems

**DOI:** 10.3390/s141019622

**Published:** 2014-10-20

**Authors:** Duk-Sun Shim, Cheol-Kwan Yang, Jae Hwan Kim, Joo Pyo Han, Yong Soo Cho

**Affiliations:** School of Electrical and Electronic Engineering, Chung-Ang University, Seoul 156-756, Korea; E-Mails: dshim@cau.ac.kr (D.-S.S.); ckyang92@empal.com (C.-K.Y.); wlswk8226@hanmail.net (J.H.K.); joopiy0628@naver.com (J.P.H.)

**Keywords:** motion sensor, accelerometer, gyroscope, geo-magnetic sensor, MEMS, rotation, displacement, mmWave, beam-tracking, blockage

## Abstract

In a millimeter wave (mmWave) communication system with transmit/receive (Tx/Rx) beamforming antennas, small variation in device behavior or an environmental change can destroy beam alignment, resulting in power loss in the received signal. In this situation, the beam-tracking technique purely based on the received signal is not effective because both behavioral changes (rotation, displacement) and environmental changes (blockage) result in power loss in the received signal. In this paper, a motion sensor based on microelectromechanical systems (MEMS) as well as an electrical signal is used for beam tracking to identify the cause of beam error, and an efficient beam-tracking technique is proposed. The motion sensors such as accelerometers, gyroscopes, and geo-magnetic sensor are composed of an attitude heading reference system (AHRS) and a zero-velocity detector (ZVD). The AHRS estimates the rotation angle and the ZVD detects whether the device moves. The proposed technique tracks a beam by handling the specific situation depending on the cause of beam error, minimizing the tracking overhead. The performance of the proposed beam-tracking technique is evaluated by simulations in three typical scenarios.

## 1. Introduction

In recent years, the millimeter wave (mmWave) band has attracted great interest for next-generation mobile communication systems that may require up to 1000 times over 4G cellular systems. It has been speculated that multi-gigabit-per-second data transmission is possible by the use of a large spectrum allocation in the mmWave frequency band and steerable beamforming antennas [1]. Highly directional beamforming antennas are necessary at both the base station (BS) and mobile station (MS) to compensate for the high attenuation in the mm-wave frequency band and to extend the transmission range [2]. With the small wavelength in mmWave frequencies, antenna arrays can be easily installed in the MS. The transmit/receive (Tx/Rx) beamforming technique using the 60 GHz unlicensed spectrum has already been standardized in IEEE 802.11ad to provide a multi-gigabit-per-second data rate [3].

Currently, an analog beamforming design is considered over digital beamforming at both the BS and MS in mmWave communication systems because multiple analog chains at mmWave frequencies are costly, and sampling an analog signal at the GHz rate consumes a substantial amount of power [4]. In practice, switched beamforming techniques with a set of pre-defined angles are being used for Tx-Rx beamforming in mmWave communication systems [5,6]. In switched beamforming systems, the maximum array gain can be obtained when Tx and Rx beams are perfectly aligned. A small misalignment between Tx and Rx beams may cause a significant loss in the received power, especially for systems with narrow beams. Therefore, beam-training in an mmWave communication system is necessary to find the best beam pair among all possible beam pairs for maximum beamforming efficiency. However, the beam-training protocol requires a large amount of training time and network resources, which are proportional to the product of the number of beams on the transmitter and receiver sides [7,8].

The beam alignment can be easily destroyed by even small variation in device behaviors such as rotation and displacement. Environmental changes such as link blockage by a foreign object may result in a significant drop in the signal power level [9,10]. Beam-tracking techniques have been investigated to track the best beam pair with a minimum overhead. However, the beam-tracking technique using the received signal power has a limitation because both behavioral changes and environmental changes result in power loss in the received signal. It is difficult to identify the cause of power loss using information purely based on the received signal [11]. In [12], a linear dynamical system (LDS) model is proposed to investigate the dynamics of signal power drops due to the errors. By using the model, it was shown that it is difficult to estimate the errors purely based on the observable quantities provided. Two active probing protocols (one-beam test and neighbor-beam test) are proposed to reveal the system state in [12]. Although the beam-training protocol can resolve such problems, it will consume significant network resources if beam-training is performed whenever the received signal experiences power loss. Techniques handling a specific type of error, such as switching to the secondary path when blockage occurs, have been proposed [13]. However, it will be more effective if we can identify the cause of power loss and handle the specific situation depending on the cause of beam error.

In this paper, an efficient beam-tracking technique for an MS in mmWave communication systems is proposed by utilizing both mechanical and electrical signals. A motion sensor based on microelectromechanical (MEMS) is employed to identify the cause of a situation change such as a rotation, displacement, or blockage. The MEMS-based motion sensor is composed of an attitude heading reference system (AHRS) [14,15], which estimates the rotation angle using a gyroscope, three-axis accelerometer, three-axis geo-magnetic sensor, and zero-velocity detector (ZVD) [16,17], which detects whether the MS moves using information from the accelerometer. The MEMS-based motion sensors are embedded in smart phones and are used in many useful applications such as games, motion detection, and navigation [18–20]. Although the uses of motion sensors, fabricated in a very small chip via MEMS technology, are increasing because of the relatively low price, no prior literature has discussed the application of motion sensors to beam-tracking in mmWave communications, to the best of our knowledge. The proposed beam-tracking technique enables us to detect the cause of the situation change and handle the specific situation depending on the cause of beam error, minimizing the tracking overhead.

The paper is organized as follows. In Section 2, we briefly investigate the required beam-tracking operation in the MS when behavioral or environmental changes occur. In Section 3, a MEMS-based motion-sensing technique including AHRS and ZVD is described. The proposed beam-tracking technique using a MEMS-based motion sensor and received electrical signal is described in Section 4. The performance of the proposed beam-tracking technique is evaluated in Section 5. Conclusions are made in Section 6.

## 2. Required Beam-Tracking Operation

**Figure 1.**
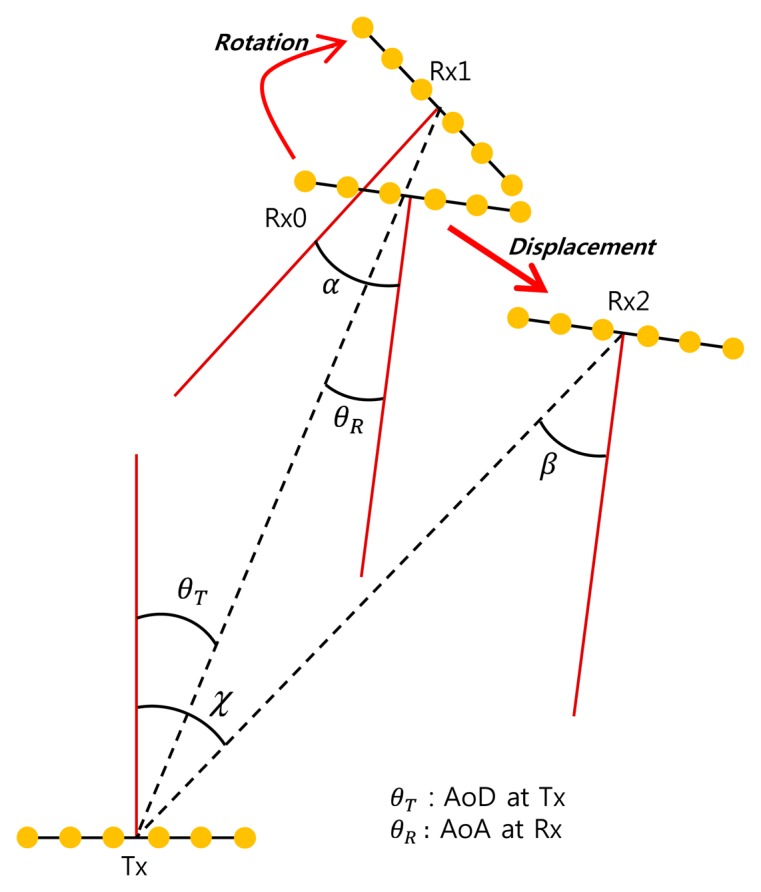
Variation in angle of departure (AoD) and angle of arrival (AoA) due to behavioral change in a mobile station (MS).

In switched beamforming systems, the received signal power may decrease when an MS rotates or moves. The primary reason for the decrease in signal power is a change in the angle of arrival (AoA) and/or angle of departure (AoD) caused by a behavioral change in the MS. Figure 1 illustrates AoD and/or AoA changes in the cases of rotation and displacement. Rx0 represents the initial position of the antenna array of the MS, where a Tx beam with the AoD (θ*_T_*) is aligned with an Rx beam with the AoA (θ*_R_*). If the MS rotates, only the value of the AoA at Rx1 changes to α, with the value of AoD being unchanged. If the MS moves, the values of AoD at Tx and AoA at Rx 2 may change to β and *χ*, respectively. The values of the AoD and AoA will not be changed when a blockage occurs. Whenever a behavioral change occurs, a beam-tracking operation needs to be performed by switching the Tx and/or Rx beams so as to maintain the Tx/Rx beam alignment. Different types of beam-tracking techniques should be implemented depending on the cause of the power reduction. Table 1 shows the changes in the AoD and AoA when a behavioral or environmental change occurs. In the case of rotation, only an Rx beam-tracking operation is required because only the value of the AoA at the MS changes. In the case of displacement, the required beam-tracking operation will be different depending on the type of displacement. If the MS stays in the same Tx beam, only an Rx beam-tracking operation is needed. If the MS does not stay in the current Tx beam, both Tx and Rx beam-tracking operations are required. If the MS is blocked by a foreign object, the beam-tracking operation over neighboring beams will not be helpful because beam misalignment is not the cause of the power reduction. It is desirable to communicate over the secondary Tx/Rx paths in such a situation if the channel experiences multipath fading [13]. The secondary path can be readily obtained from the beam-training protocol at the initialization stage.

## 3. A MEMS-Based Motion Sensing Techniques for Beam-Tracking

This section describes a MEMS-based motion sensing technique required for beam-tracking in mmWave communication systems.

### 3.1. Coordinate Transformation

**Figure 2.**
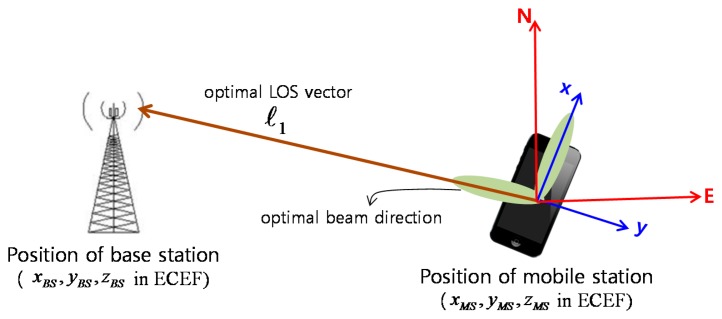
Coordinate frames of base station (BS) and MS for finding an optimal beam direction.

Euler angles are frequently used to specify the angular orientation of one coordinate system relative to another. A series of three ordered right-handed rotations is necessary in the sequence of yaw, pitch, and roll. Euler angles are defined in the north-east-down (NED) navigation frame, where the *x*-axis indicates the north direction, the *y*-axis indicates the east direction, and the *z*-axis indicates the downward direction perpendicular to the horizon. The navigation frame is used to define the attitude (roll, pitch, yaw) of a vehicle or a device. Figure 2 shows coordinates systems of a BS and an MS for beam-tracking. The line-of-sight (LoS) vector, ℓ, from the MS to BS, and the attitude of the MS need to be known to find the optimal beam pair between the BS and MS. The LoS vector $\ell_{1}^{e}$ denoting the optimal beam direction expressed in the Earth-Centered Earth-Fixed (ECEF) frame is transformed to the LOS vector $\ell_{1}^{e}$ in the body frame as in Equation (1), where vector $\ell_{1}^{e}$ can be calculated from Equation (2). The ECEF frame has its origin at the center of the Earth and rotates with the same angular rate of the Earth. The body frame has its origin at the center of gravity of the MS with the *x*-axis in the forward direction and the *z*-axis in the downward direction.

(1) $${\ell_{1}}^{b}=C_{l}^{b}C_{e}^{l}{\ell_{1}}^{e}$$

(2) $${\ell_{1}}^{e}={\left[\begin{array}{lll} x_{MS}-x_{BS} & y_{MS}-y_{BS} & z_{MS}-z_{BS} \end{array}\right]}^{T}$$

where (*x_MS_*, *y_MS_*, *z_MS_*,)and (*x_BS_*, *y_BS,_* *z_BS_*)denote the position vectors of the MS and BS, respectively, expressed in the ECEF frame.

The direction cosine matrix from the ECEF frame to the NED navigation frame (*l*), $C_{e}^{l}={\left(C_{l}^{e}\right)}^{T}$, is expressed with the position of the BS, latitude φ, and longitude λ, as in Equation (3). The direction cosine matrix from the NED navigation frame to the body frame, $C_{l}^{b}={\left(C_{b}^{l}\right)}^{T}$, is expressed with the attitude of the BS, roll (*ϕ*), pitch (θ), and yaw (ψ), as in Equation (4) [21].

(3) $$C_{l}^{e}=[\begin{array}{ccc} -\sin\lambda & -\sin\varphi \cos\lambda & \cos\varphi \cos\lambda \\ \cos\lambda & -\sin\varphi \sin\lambda & \cos\varphi \sin\lambda \\ 0 & \cos\varphi & \sin\varphi \end{array}]$$

(4) $$C_{b}^{l}=[\begin{array}{ccc} \cos\theta \cos\psi & -\cos\phi \sin\psi +\sin\phi \sin\theta \cos\psi & \sin\phi \sin\psi +\cos\phi \sin\theta \cos\psi \\ \cos\theta \sin\psi & \cos\phi \cos\psi +\sin\phi \sin\theta \sin\psi & -\sin\phi \cos\psi +\cos\phi \sin\theta \sin\psi \\ -\sin\theta & \sin\phi \cos\theta & \cos\phi \cos\theta \end{array}]$$

To find the LoS vector $\ell_{1}^{e}$, positions of the BS and MS should be known in advance. If the position information of the BS is available at the MS, and there always exists an LoS vector, the optimal beam direction can be found by Equation (1). However, when the position information of the BS is not available at the MS or only a non-line-of-sight (NLoS) channel exists, Equation (1) cannot be used. In a NLoS (multipath) channel environment, the signal transmitted from the BS is reflected by an object (building) and received by the MS. In this situation, the optimal beam direction for the MS will be the vector between the object and MS. Because the initial vector $\ell_{1}^{b}$ can be obtained through the initial beam-training protocol in this situation, the beam direction in the next frame can be obtained iteratively as shown in Equation (5).

(5) $$\begin{array}{ll} \ell_{1}^{b(k+1)}=C_{b(k)}^{b(k+1)}\ell_{1}^{b(k)}, & k=0,1,2,\cdots \end{array}$$

where $C_{b(k)}^{b(k+1)}$ is the coordinate transformation matrix from the *k*-th body frame to (*k* + 1)-th body frame as in Equation (6).

(6) $$C_{b(k)}^{b(k+1)}=[\begin{array}{ccc} \cos\Delta \theta_{k}\cos\Delta \psi_{k} & -\cos\Delta \phi_{k}\sin\Delta \psi_{k}+\sin\phi \sin\Delta \theta_{k}\cos\Delta \psi_{k} & \sin\Delta \phi_{k}\sin\Delta \psi_{k}+\cos\Delta \phi_{k}\sin\Delta \theta_{k}\cos\Delta \psi_{k}\\ \cos\Delta \theta_{k}\sin\Delta \psi_{k} & \cos\Delta \phi_{k}\cos\Delta \psi_{k}+\sin\phi \sin\Delta \theta_{k}\sin\Delta \psi_{k} & -\sin\Delta \phi_{k}\sin\Delta \psi_{k}+\cos\Delta \phi_{k}\sin\Delta \theta_{k}\cos\Delta \psi_{k}\\ -\sin\Delta \theta_{k} & \sin\Delta \phi_{k}\cos\Delta \theta_{k} & \cos\Delta \phi_{k}\cos\Delta \theta_{k} \end{array}]$$

where Δ*ϕ_k_* = *ϕ_k_*_+1_−*ϕ_k_*, Δθ*_k_* = θ*_k_*_+1_−θ*_k_*, and Δψ*_k_* = ψ*_k_*_+1_−ψ*_k_* denote the differences in Euler angles between the *k*-th and (*k*+1)-th time sequences.

**Figure 3.**
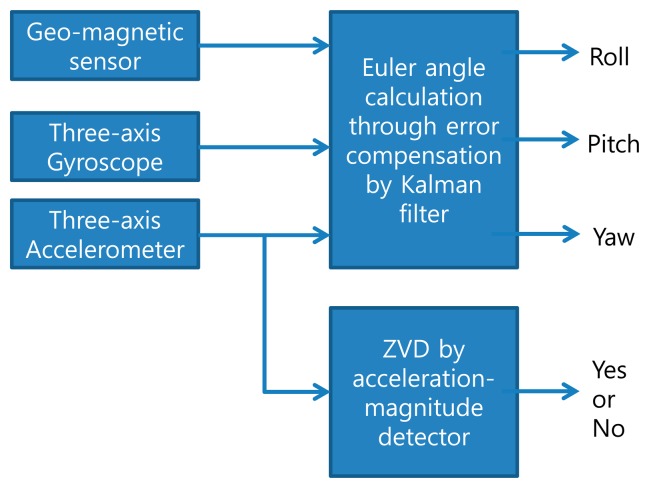
A block diagram of a motion sensor for beam-tracking.

Two types of behavioral change, rotation and displacement, are considered. In order to distinguish different types of device behavior, the following information should be obtained. Firstly, the information regarding roll, pitch, and yaw should be obtained. Secondly, whether the MS has moved needs to be checked. We use an AHRS, which provides the roll, pitch, and yaw of the MS using MEMS devices (a geo-magnetic sensor, three-axis gyroscope, and accelerometer), and a ZVD, which discerns whether the MS has moved using information only from the accelerometer. Figure 3 shows a block diagram of a motion sensor composed of an AHRS and ZVD. The following two sub-sections describe the AHRS and ZVD in detail.

### 3.2. Attitude Heading Reference System (AHRS)

Two different types of methods can be considered in obtaining attitude information. Firstly, an accelerometer and a geo-magnetic sensor embedded in the MS can be used. The gravity output from the accelerometer provides roll and pitch information, and the geo-magnetic sensor provides yaw information. This method maintains a certain degree of accuracy regardless of the duration of the measurement period. However, each sensor individually provides rather inaccurate attitude information, particularly in the case of movement. Secondly, a MEMS gyroscope can also be used to obtain attitude information. The accuracy of attitude information is relatively high for a short period of time in this method, but errors are accumulated as time increases because the attitude information is obtained by integrating the angular rate, which is the output of the gyroscope.

An AHRS algorithm, which combines the two methods above, can estimate the attitude information with significantly higher accuracy. The AHRS algorithm uses a Kalman filter after transforming the attitude information of the roll, pitch, and yaw to quaternions [14,15]. The Kalman filter provides optimal estimation performance for linear models. While the dynamic equation of the roll, pitch, and yaw is highly nonlinear, the dynamic equation of quaternions is a linear differential equation, as shown in Equation (10), given the initial condition and the forcing functions of *p*, *q*, and *r*. The quaternion defined by the rotation vector between the body frame and the navigation frame has four components, as in Equation (7).

(7) $$\vec{q}={\left[\begin{array}{llll} q_{0} & q_{1} & q_{2} & q_{3} \end{array}\right]}^{T}$$

The quaternion *q⃗* can be expressed with the attitude information, *ϕ*, θ, and ψ, as in Equation (8).

(8) $$\begin{array}{l} q_{0}=\cos\frac{\varphi }{2}\cos\frac{\theta }{2}\cos\frac{\psi }{2}+\sin\frac{\varphi }{2}\sin\frac{\theta }{2}\sin\frac{\psi }{2}\\ q_{1}=\sin\frac{\varphi }{2}\cos\frac{\theta }{2}\cos\frac{\psi }{2}-\cos\frac{\varphi }{2}\sin\frac{\theta }{2}\sin\frac{\psi }{2}\\ q_{2}=\cos\frac{\varphi }{2}\sin\frac{\theta }{2}\cos\frac{\psi }{2}+\sin\frac{\varphi }{2}\cos\frac{\theta }{2}\sin\frac{\psi }{2}\\ q_{3}=\cos\frac{\varphi }{2}\cos\frac{\theta }{2}\sin\frac{\psi }{2}-\sin\frac{\varphi }{2}\sin\frac{\theta }{2}\cos\frac{\psi }{2} \end{array}$$

The attitude information, *ϕ*, θ, and ψ, can be obtained from the quaternion *q⃗* as in Equation (9).

(9) $$C_{b}^{l}=[\begin{array}{ccc} q_{0}^{2}+q_{1}^{2}-q_{2}^{2}-q_{3}^{2} & 2(q_{1}q_{2}-q_{0}q_{3}) & 2(q_{1}q_{3}+q_{0}q_{2})\\ 2(q_{1}q_{2}+q_{0}q_{3}) & q_{0}^{2}-q_{1}^{2}+q_{2}^{2}-q_{3}^{2} & 2(q_{2}q_{3}-q_{0}q_{1})\\ 2(q_{1}q_{3}+q_{0}q_{2}) & 2(q_{2}q_{3}+q_{0}q_{1}) & q_{0}^{2}-q_{1}^{2}-q_{2}^{2}+q_{3}^{2} \end{array}]$$

$$\varphi =\tan^{-1}(\frac{C_{b}^{l}(3,2)}{C_{b}^{l}(3,3)}),\theta =-\tan^{-1}(\frac{C_{b}^{l}(3,1)}{\sqrt{C_{b}^{l}{\left(3,2\right)}^{2}+C_{b}^{l}{\left(3,3\right)}^{2}}}),\psi =\tan^{-1}(\frac{C_{b}^{l}(2,1)}{C_{b}^{l}(1,1)})$$

The operation of the AHRS can be summarized as follows. First, the differential equations of quaternions are formed as in Equation (10), where quaternion *q⃗_G_* is obtained by Euler angles calculated from the three-axis gyroscopes outputs.

(10) $${\dot{\vec{q}}}_{G}=\frac{1}{2}\Omega {\vec{q}}_{G}+w$$

where *w* is the process noise and Ω is the matrix consisting of three-axis gyroscope outputs, *p*, *q* and *r*.

(11) $$\Omega =[\begin{array}{llll} 0 & -p & -q & -r\\ p & 0 & r & -q\\ q & -r & 0 & p\\ r & q & -p & 0 \end{array}]$$

Next, the yaw angle is calculated from the output of the geo-magnetic sensor, and the roll and pitch angles are calculated from the output of the three-axis gyroscope. The quaternion *q⃗_R_* is formed by the information (roll, pitch, and yaw) obtained from Equation (8). Then, the measurement equation of the Kalman filter is formed as in Equation (12).

(12) $$z={\vec{q}}_{R}-{\vec{q}}_{G}$$

The strength of the earth's magnetic field is as weak as 0.4 Gauss, and thus it easily receives interference from ambient magnetic fields. For most applications, the motion of character 8 is used to remove the ambient interference. To do this, it is necessary to initially employ a detection scheme to check whether the interference is present. In the AHRS algorithm used in this paper, we detect the ambient interference signal using the magnitude of the azimuth residual in the fusion Kalman filter. If the magnitude of the azimuth residual increases abruptly in a short time, then interference of the magnetic field is considered to have occurred, and the compensation message is sent to the users.

In general, there are two methods of compensating for the bias of inertial sensors. One method is to use the averaged value of measurements for compensation. The other method is to use a high-pass filter to remove the bias. Those two methods have strengths and weaknesses depending on the characteristics of the dynamic environment. We use the first method in this paper because it is appropriate for a case with a small dynamic range.

### 3.3. Zero-Velocity Detector (ZVD)

A ZVD provides a criterion regarding whether an inertial measuring unit (IMU) moves. Four different methods such as acceleration moving variance (MV) detector, acceleration magnitude (MAG) detector, angular rate energy (ARE) detector, and stance hypothesis optimal detection (SHOE) detector, are proposed in [16,17]. In these methods, the outputs of inertial sensors, gyroscopes and accelerometers, are used for ZVD. The measurements with the accelerometer and gyroscope, $z_{n}^{a}\equiv {\left\{y_{k}^{a}\right\}}_{k=n}^{n+W-1}$ and $z_{n}^{\omega }\equiv {\left\{y_{k}^{\omega }\right\}}_{k=n}^{n+W-1}$, are used to detect the movement of the IMU. Here, *W* denotes the size of the measurement. In addition, $y_{k}^{a}\in R^{3}$ and $y_{k}^{\omega }\in R^{3}$, *k* ∈ *N*, are the *k*-th measurement vectors of the specific force and angular velocity, respectively. The superscripts, *a* and *ω*, denote the acceleration and angular velocity, respectively. The detection algorithm has a decision rule similar to binary hypothesis testing, as in Equation (13).

(13) $$T(z_{n}^{a},z_{n}^{\omega })<\gamma$$

where $T(z_{n}^{a},z_{n}^{\omega })$ is the test statistic of the detector and γ is the detection threshold. Gyroscopes are used for ARE and SHOE detectors. One drawback of using gyroscopes for ZVD is that the MS may be considered to be moving even though it stays still, particularly when there is a large attitude change. Meanwhile, with the MV and MAG detectors using an accelerometer, the MS is always detected as stationary when it stays still, even with a large attitude change.

We use the MAG detector, which provides similar performance to the MV detector with less computational complexity. The MAG detector detects whether the IMU is stationary depending on the closeness of the measured specific force to gravity *g*, and uses the test statistic in Equation (14) [16].

(14) $$T(z_{n}^{a},z_{n}^{\omega })=\frac{1}{\sigma_{a}^{2}W}\overset{n+W-1}{\underset{k=n}{\text{∑}}}{\left(\|y_{k}^{a}\|-g\right)}^{2}$$

where $\sigma_{a}^{2}\in R$, the variance of the measurement noise of the accelerometer, adjusts the range of the test statistic without affecting the performance of the MAG detector. Here, *_W_* and γ are the only tuning parameters of the MAG detector.

## 4. Proposed Beam-tracking Technique Using Motion Sensor

The proposed beam-tracking technique minimizes the beam-tracking overhead (network resources and processing time) by precisely estimating the cause of a beam error in the MS. In the proposed beam-tracking technique, the electrical signal, initial beam index, and motion sensor output are used as inputs. When the MS rotates, the rotation angle is estimated by the AHRS. The angle of the initial Rx beam and the angle estimated by the AHRS are inputted into an angle variation calculator, which estimates the angle difference and derives the Rx beam index to be updated. The ZVD detects whether the MS moves. The received electrical signal power, angle variation calculator output, and ZVD output are inputted into a situation detector, where the cause of the beam error is detected. Beam-tracking operation corresponding to the specific situation (rotation, displacement, and blockage) is performed accordingly.

**Figure 4.**
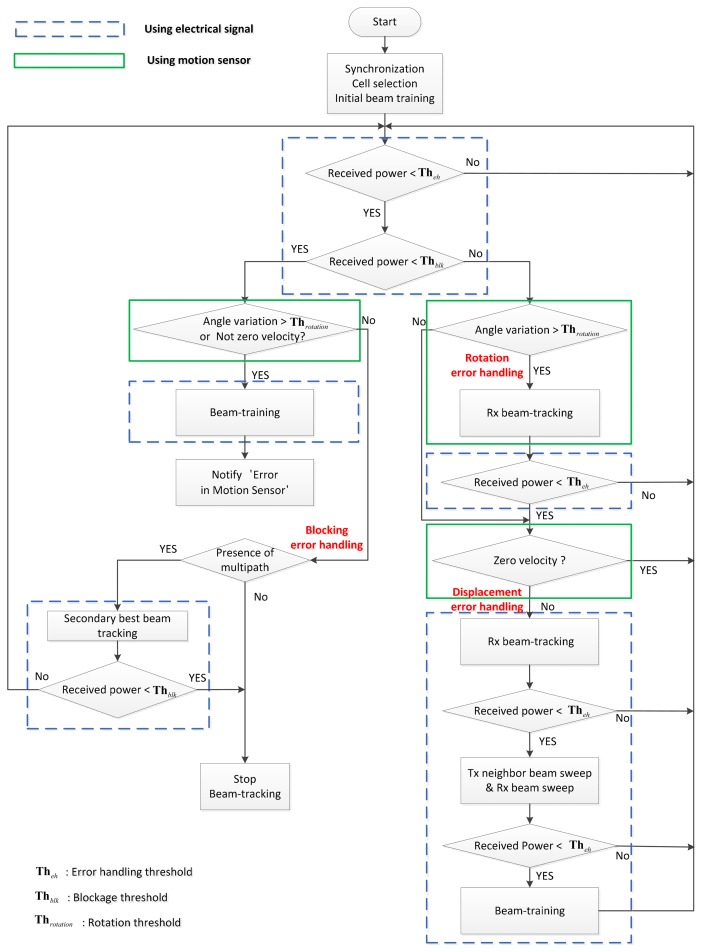
A flowchart of the proposed beam-tracking technique.

Figure 4 shows a flowchart of the proposed beam-tracking technique. In this figure, the block with a dotted line is performed with an electrical signal, whereas the block with a solid line is performed with a signal from the motion sensor. The error handling procedures for rotation and displacement are provided on the right-hand side, and the procedure for blockage is provided on the left-hand side in this figure. It is assumed that the initial synchronization, cell searching, and beam-training protocol have been completed. A beam-tracking procedure begins when the power of the received signal is below *Th_eh_* (threshold for error handling). If the power is lower than *Th_eh_* but higher than *Th_blk_* (threshold for blocking), the procedure checks whether the MS is rotated. The possibility of rotation is examined first because rotation occurs most frequently and can be resolved rapidly only by the Rx tracking operation. Angle variation, *i.e.*, deviation in the AoA of an Rx beam, is measured by the motion sensor. If the value of the angle variation is greater than *Th_rotation_* (threshold for rotation), the Rx beam tracking operation in Equation (6) is performed with the updated beam index obtained by the angle variation calculator. Once the power becomes greater than *Th_eh_* after the Rx beam-tracking operation, it returns to the initial stage. If the power is still lower than *Th_eh_* after Rx beam-tracking, the test of displacement will be carried out. If the output of the ZVD indicates that the MS is moving, the Rx beam-tracking operation is performed first for the case where the MS stays in the same Tx beam. If the power is still lower than *Th_eh_* after the Rx beam-tracking, Tx beam-tracking is performed over neighboring Tx beams.

If the power of the received signal is lower than *Th_blk_* at the initial stage, the procedure checks whether blockage has occurred. The block on the left-hand side checks whether the power drop is caused by a behavioral change or environmental change. If no behavioral change (rotation, displacement) is detected, it will be assumed that the power drop is caused by an environmental change (blockage). In the presence of a multipath channel, the blockage error handling procedure switches the beam to the secondary Tx/Rx paths, which can be made available through the initial beam-training process. If no secondary path is available, the beam-tracking procedure is terminated. If the power of the received signal is lower than *Th_blk_* and a behavioral change is detected by the AHRS or ZVD, there is a high probability of malfunction in the motion sensor. In this case, the state of malfunction in the motion sensor is communicated by sending the alarm message “error in motion sensor”, after finishing beam-training protocol with only an electrical signal.

## 5. Performance Analysis of the Proposed Beam-Tracking Technique

In this section, computer simulations are performed to evaluate the performance of the proposed beam-tracking technique. Three scenarios are considered. For 4 s, it is assumed that rotation (Scenario 1), displacement (Scenario 2), and blockage (Scenario 3) take place in sequence. In order to evaluate the performance of the proposed approach for each case, it is assumed that these changes (behavioral or environmental) do not occur at the same time. In all scenarios, it is assumed that the person is holding the device (MS). In Scenario 1, the MS rotates clockwise during the first 0.45 s, stands still for the next 0.1 s, and rotates counterclockwise during the last 0.45 s. It is assumed that the MS rotates only in the angular direction of yaw. It is also assumed that the person holding the device does not block the main beam direction. In Scenario 2, the MS moves for 2 s in total: To the north for the first second, and to the south for next second. It is assumed that the BS is located to the west of the MS, and the distance between the BS and MS is 50 m. In Scenario 3, it is assumed that blockage occurs because of the person or another pedestrian and the secondary path is available. The mean and RMS values of the power drop due the blockage are assumed to be −17 dB and 2 dB, respectively [10]. The mean drop time in the blockage is assumed to be 93 ms. Major parameters for sensor errors are provided in Table 2. It is assumed that the measurement rate of the accelerometer and gyroscope is 1 KHz, and the measurement rate of the geo-magnetic sensor is 100 Hz. The power of the received electrical signal is measured every 1 ms. The following parameters are used for beam tracking. An AWGN channel with *E_b_*/*N*_0_ of 20 dB is used. The numbers of antennas and beams are 16 and 32, respectively. The values of *Th_eh_* and *Th_bk_* are −2 dB and −17 dB, respectively. *Th_rotation_* is set to 5.625°. The error handling delay is assumed to be 0.3 ms. A uniform linear array (ULA) is used at the BS, and a uniform circular array (UCA) is used at the MS.

**Figure 5.**
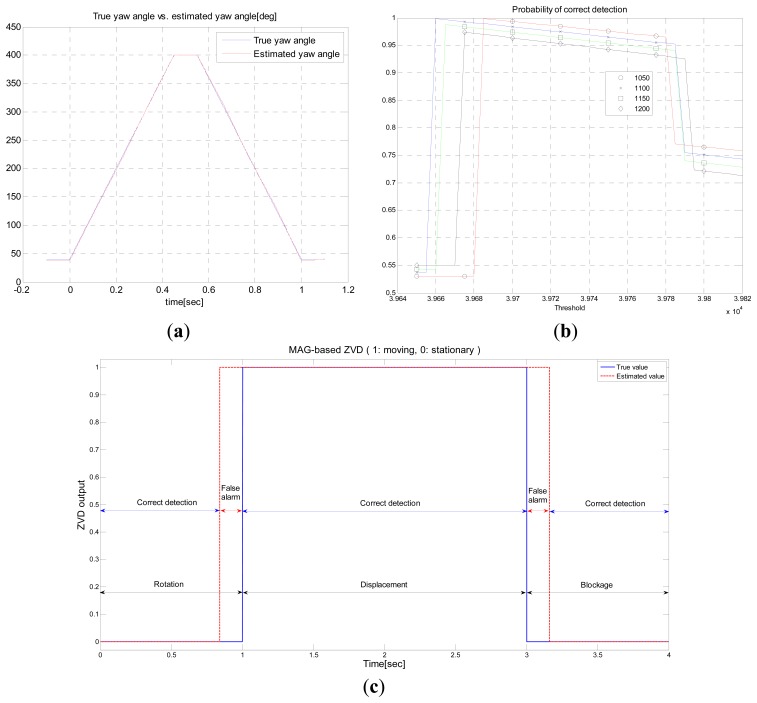
Performance of AHRS (**a**) Yaw angle estimated by AHRS in Scenario 1; (**b**) Probability of correct detection for the MAG-based ZVD (**c**) Performance of the MAG-based ZVD for three different scenarios.

Figure 5 shows the performance of the AHRS. The yaw angle estimated by the AHRS in Scenario 1 is shown in Figure 5a. From this figure, one can observe that the estimated angle is close to the true value (estimation error is approximately 1.5°–2.0°). Note that an accurate estimate is possible even when the rotational speed of the MS is high. In this simulation, the rotational speed is 800 °/s. Figure 5b shows the performance of the MAG-based ZVD. The probability of correct detection in the MAG-based ZVD is evaluated in terms of the window size *_W_* and threshold value γ. The range of window sizes is 1050–1200, and the range of the threshold value γ is 39,650–39,820. The range of window and detection threshold values are determined as follows: For the scenarios used in this paper, MEMS-level IMU sensor data are generated using the MATLAB INS Toolbox, and the threshold is determined using a Monte Carlo simulation with the generated data. Monte Carlo simulations are performed for various combinations of window size and threshold for given scenarios. The optimal window size and threshold value are determined based on correct alarm probability.

From the simulation results, one can observe that the probability of correct detection is the highest when *W* = 1050. The widest span of the threshold value at 39,660–39,785 is obtained at *W* = 1100 while maintaining the probability of correct detection of 0.94. Meanwhile, in the case of *W* at 1150 and 1200, the probability of correct detection is relatively low, and the performance is good only for narrow range of threshold values. Figure 5c shows the performance of the MAG-based ZVD with a window size of 1100 and a threshold value of 39,750 for Scenarios 1–3. The MAG-based ZVD shows good performance with the probability of correct detection close to 0.95. It is noted that false alarms occur during the period of 0.85 s–1.0 s and 3.0 s–3.15 s when the MS moves after stopping and stops after moving, respectively. The reason for the false alarm is that two different types of signals, a signal received while moving and a signal received while stationary, are mixed together when 1100 samples are used to calculate the test statistic, $T(z_{n}^{a},z_{n}^{\omega })$.

**Figure 6.**
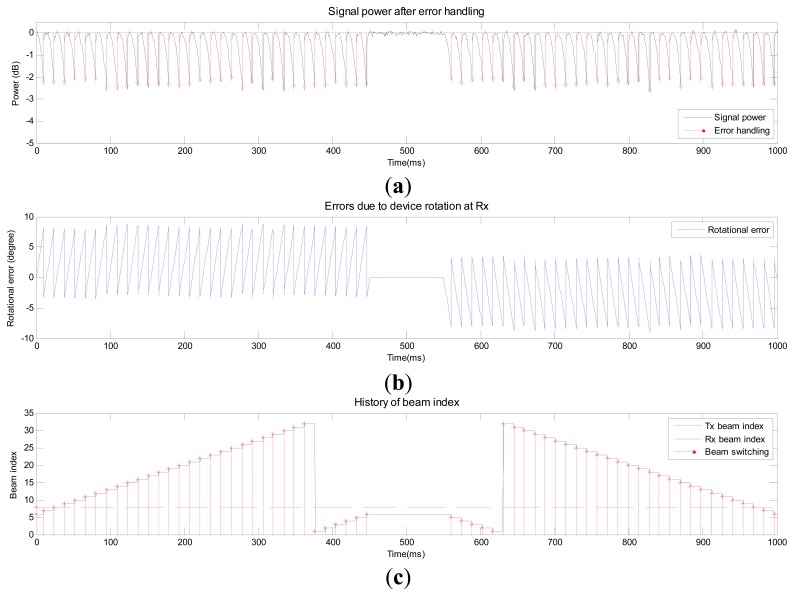
Performance of the proposed beam-tracking technique in Scenario 1.

**Figure 7.**
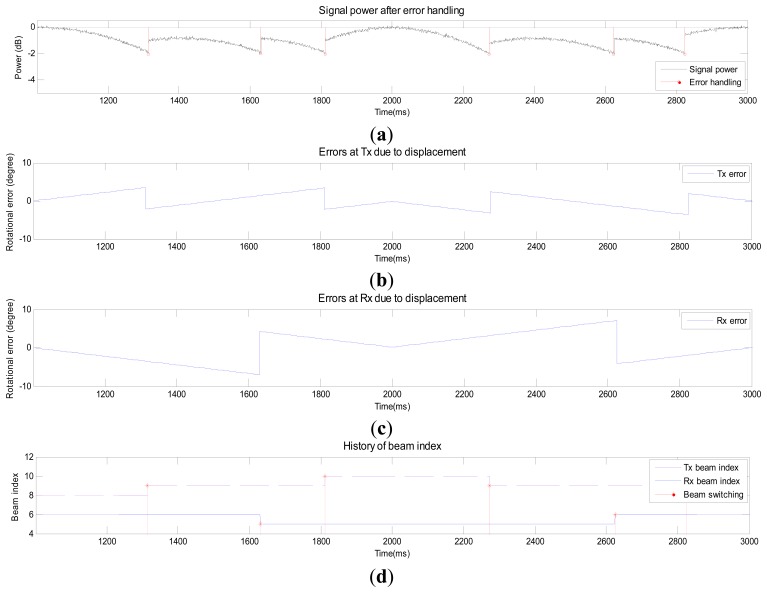
Performance of the proposed beam-tracking technique in Scenario 2.

**Figure 8.**
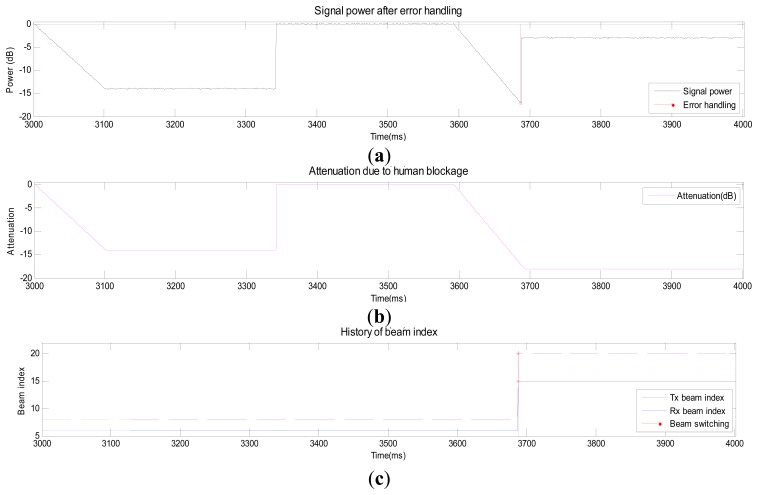
Performance of the proposed beam-tracking technique in Scenario 3.

Figures 6, 7 and 8 show the performance of the proposed beam-tracking technique in Scenarios 1–3, respectively. Figure 6a shows the signal power after error handling when the MS rotates. Figure 6b and c shows the error at Rx and history of beam index (Tx beam and Rx beam), respectively. In Figure 6a, the instants when the beam-tracking operation is performed (<*Th_eh_*) are marked as “o”. In addition, the instants when the Rx beam is switched are marked as “*” in Figure 6c. From Figure 6b, one can observe that the angular error drops after the Rx beam is switched to a new beam corresponding to the output of the angle variation calculator. The index of the Tx beam in Figure 6c is not changed in Scenario 1.

Figure 7a shows the signal power after error handling when the MS moves. Figure 7b and c shows angular errors in the AoD and AoA, respectively. The error in AoD increases while the error in AoA decreases as the MS moves north. The angular error drops after the Tx or Rx beam is switched to a new Tx or Rx beam. The index of the Tx beam in Figure 7d starts with #8 and switches to #10 during the first second. The index reverts back to #8 during the last second. Figure 8a shows the signal power after error handling when a blockage occurs. Signal attenuation due to human blockage is shown in Figure 8b when no blockage error handling procedure is used. No action is taken until the signal power reaches the value of *Th_blk_* as can be observed in Figure 8a and c. During this period, no beam tracking operation is performed because the situation (blockage) can be detected by using the information (ZVD, AHRS, and received signal power). Once the received signal power becomes lower than *Th_blk_*, the Tx and Rx beams are switched to the secondary path (#20 and #15) as can be seen from Figure 8c.

Next, the proposed beam-tracking technique is compared with a “conventional technique” purely based on the electrical signal in terms of the number of beam switches, which is directly related with the processing time and power consumption required for beam-tracking. Table 3 shows the number of beam switches required for rotation, displacement, and blockage. Here, $N_{Rx}^{b}$, $N_{Tx}^{b}$, and $N_{Tx}^{nb}$ denote the number of Rx beams, number of Tx beams, and number of neighboring beams for Tx beam-tracking, respectively. In the case of rotation, an Rx beam is directly switched to the position of the best beam with the aid of AHRS in the proposed technique, whereas the Rx beam sweep is needed in the conventional technique. In the case of displacement, the same number of beam switches is required for both techniques. In the case of blockage, unnecessary beam-tracking can be avoided in the proposed technique because blockage can be easily detected with the aid of ZVD. However, in the conventional technique, the beam-tracking procedure should be repeated until the received power becomes lower than *Th_blk_*. Table 4 shows the number of beam switches required for three different scenarios. The numbers of error handlings that occurred in Scenarios 1–3 were 70, 6, and 410, respectively. It is assumed that $N_{Rx}^{b}=32$, $N_{Tx}^{b}=32$ and $N_{Tx}^{nb}=4$. The numbers of Tx and Rx beam switches required for the proposed technique are significantly reduced to 10.4% and 1.4% of the numbers for the conventional technique, respectively.

## 6. Conclusions

In this paper, an efficient beam-tracking technique for an MS in mmWave communication systems is proposed using MEMS-based motion sensors. It was shown by simulation that the angle of device rotation can be accurately estimated by the AHRS consisting of accelerometer, gyroscope, and geo-magnetic sensor, and the device movement can be detected by the MAG-based ZVD with appropriate parameters. In the case of rotation, the proposed technique was shown to track the beam efficiently by switching directly to the position of the best Rx beam with the aid of AHRS, resulting in a significant reduction in the length of the beam search. In the case of displacement, the proposed technique was shown to track the Rx beam and/or Tx beam using the received electrical signal. In the case of blockage, the proposed technique was shown to avoid unnecessary beam-tracking by detecting the blocking status with the aid of the MAG-based ZVD. Overall, the proposed technique was shown to track the beam efficiently by detecting the cause of the situation change and handling the specific situation depending on the cause of beam error, minimizing the beam-tracking overhead.

## Figures and Tables

**Table 1.** Required beam-tracking operation when a behavioral or environmental change occurs.

**Item**	**Beam-Tracking**	**Rotation**	**Displacement**	**Blocking**
Characteristics of change	θ*_T_* (Tx AoD)	No change	Change	No change
	θ*_R_* (Rx AoA)	Change	Change	No change

Required beam-tracking	Rx beam-tracking	Necessary	Necessary	Not necessary
	Tx beam-tracking	Not necessary	Necessary	Not necessary
	Secondary beam	Not necessary	Not necessary	Necessary

**Table 2.** Major parameters for sensor errors.

**Item**	**Accelerometer**	**Gyroscope**	**Geo-Magnetic Sensor**
Bias	125 mg	100 deg/hr	1,250,000 ppm
Scale factor error	50,000 ppm	60,000 ppm	250,000 ppm
Noise	5 mg	1 deg/hr	10,000 ppm

(g: Gravity acceleration).

**Table 3.** Number of beam switches required for beam-tracking.

		**Rotation**	**Displacement**	**Blockage**
Conventional technique	Tx	0	$N_{Tx}^{nb}$	$N_{Tx}^{nb}$
	Rx	$N_{Rx}^{b}$	$N_{Rx}^{b}(N_{Tx}^{nb}+1)$	$N_{Rx}^{b}(N_{Tx}^{nb}+1)$

Proposed technique	Tx	0	$N_{Tx}^{nb}$	0
	Rx	0	$N_{Rx}^{b}(N_{Tx}^{nb}+1)$	0

**Table 4.** Number of beam switches required for beam-tracking in three different scenarios.

		**Scenario 1**	**Scenario 2**	**Scenario 3**	**Total**
Conventional technique	Tx	0	192	1,640	1,832
	Rx	2,240	960	65,600	68,800

Proposed technique	Tx	0	192	0	192
	Rx	0	960	0	960
